# Genetics of Congenital Heart Defects: The NKX2-5 Gene, a Key Player

**DOI:** 10.3390/genes7020006

**Published:** 2016-01-23

**Authors:** Ill-Min Chung, Govindasamy Rajakumar

**Affiliations:** Department of Applied Bioscience, College of Life and Environmental Science, Konkuk University, Seoul 05029, Korea; imcim@konkuk.ac.kr

**Keywords:** congenital heart defect, mutations, genetic testing, NKX2-5 gene, inheritance

## Abstract

Congenital heart defects (CHDs) represent the biggest fraction of morbid congenital anomalies worldwide. Owing to their complex inheritance patterns and multifactorial etiologies, these defects are difficult to identify before complete manifestation. Research over the past two decades has established firmly the role of genetics in the development of these congenital defects. While syndromic CHDs are more straightforward, non-syndromic CHDs are usually characterized by multiple mutations that affect intricate inter-connected developmental pathways. Knock-out and gene expression studies in mice and other genetic models have been performed to elucidate the roles of these implicated genes. Functional analysis has not been able to resolve the complete picture, as increasingly more downstream effects are continuously being assigned to CHD mutant factors. NKX2-5, a cardiac transcription factor, has received much attention for its role in cardiac dysmorphogenesis. Approximately 50 different mutations in this gene have been identified to date, and only a few have been functionally characterized. The mutant NKX2-5 factor can regulate a number of off-targets downstream to facilitate CHD development. This review summarizes the genetic etiology of congenital heart defects and emphasizes the need for NKX2-5 mutation screening.

## 1. Introduction

Congenital heart defects (CHDs) are defects in the structure of the heart and great vessels that occur at birth. Errors in septation, proper patterning of the great vessels, and valve formation are the most common aberrations in cardiac development that lead to the majority of CHDs seen. Previously, most deaths resulting from CHDs occurred during infancy. Now, with great advancements in prenatal diagnosis and corrective strategies, the number of infantile deaths has declined, and more than 3/4 of children with congenital malformations survive into adulthood, including those with complex abnormalities [1].

**Figure 1:**
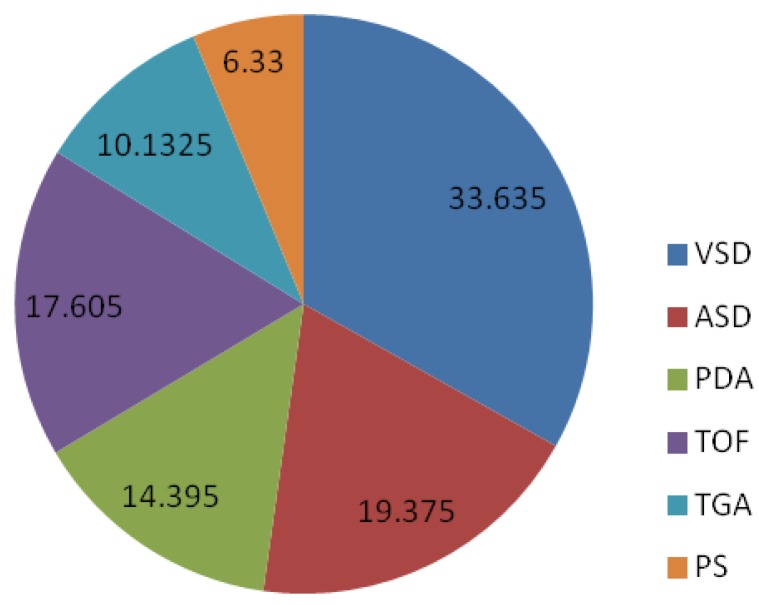
Incidence of different types of congenital heart defects. VSD, ventricular septal defect; PS, pulmonary stenosis; PDA, patent ductus arteriosus; ASD, atrial septal defect; TGA, transposition of the great arteries.

Although CHDs are normally found in the pediatric age group (0–15 years), it is not uncommon to see adults with corrected or uncorrected CHDs. Some patients may not manifest any symptoms characteristic of congenital heart diseases, and hence, the condition might not be noticed until complications arise later. The rate of affected individuals who survive into adulthood varies with the severity of the disease; these figures vary from 98% of patients with a mild CHD, 96% of patients with a moderate CHD, and only 56% of patients with a severe CHD [2]. Adults who previously underwent corrective procedures for CHDs also need to be monitored, as they are at risk for late complications such as arrhythmias, endocarditis, and heart failure, leading to the need for additional surgery [3]. Figure 1 shows the incidence of different types of congenital heart defects in Asia [4,5,6,7].

The past decade has witnessed a significant amount of research aiming to establish causative factors for various CHDs. Genetic mutations and polymorphisms have been speculated to be the prime cause, and numerous studies to reveal these variations have been undertaken. Models have been proposed to define the inheritance patterns seen in familial CHDs and the genetic factors that contribute to familial and sporadic CHD types. Molecular pathways that are at the crux of the cardiogenesis process have been delineated. It has come to light that the molecules in these pathways promote spatial and temporal gene expression patterns [8,9], the disruption of which triggers abnormal development.

This article aims to elucidate the role of genetic factors in congenital cardiac anomalies and emphasizes the need for genetic testing of affected individuals.

## 2. Inheritance Pattern of CHDs

The genetics of CHDs is highly heterogeneous. CHD mutations could be familial or sporadic in nature. Familial CHD mutations may occur as autosomal dominant, autosomal recessive, or X-linked traits. These mutations are highly penetrant and result in a variety of clinical manifestations. From an evolutionary standpoint, CHD mutations should be eliminated through negative selection, as they result in reduced reproductive fitness and early mortality. Therefore, if mutations that contribute to the CHD prevalence of a population are dominant or X-linked, they will mostly be *de novo* in nature [10]. In addition, recurrence rates are expected to be high in the offspring of cases with sporadic dominant mutations. However, studies have shown otherwise, challenging the notion that dominant *de novo* mutations are a major contributor to CHDs.

Autosomal recessive and polygenic variants serve as alternative genetic models for the population prevalence of CHD. The risk for a wide range of CHD phenotypes worldwide is increased by two- or threefold by parental consanguinity. A study carried out in the South Indian population revealed that two CHD subtypes, atrial septal defect (ASD) and patent ductus arteriosus (PDA), are frequently associated with consanguinity. Such data in turn indicate that the mutations that contribute to CHD prevalence in a population follow a recessive genetic model [11].

## 3. Etiological Factors

The major causes of CHDs include the following: (a) chromosomal disorders and single gene disorders, constituting 8%; (b) environmental teratogens, constituting 2%; and (c) a complex, multifactorial etiology, constituting 90%.

### 3.1. Chromosomal Disorders

The association of CHDs with chromosomal syndromes has led to the classification of these defects into (a) Syndromic CHDs and (b) Non-syndromic or Isolated CHDs. Syndromic CHDs are associated with chromosomal aneuploidy, abnormal chromosomal structure, and single gene mutations. The first recognized genetic cause of CHDs was chromosomal aneuploidy. The syndromes that usually accompany most congenital heart defects (Table 1) include:

(1) **Trisomy 21**: Also called Down’s syndrome, this condition is characterized by the presence of an extra chromosome 21 and usually occurs with heart defects. Approximately 40%–50% of all Trisomy 21 children are affected by a congenital heart defect. Atrioventricular septal defects (AVSDs) constitute a majority of these cases [12].
(2) **Trisomy 18**: Also called Edward’s syndrome, this condition is characterized by the presence of an extra copy of chromosome 18. Of all trisomy 18 births, 80%–100% are associated with a CHD. ASDs, ventricular septal defects (VSDs), and patent ductus arteriosus (PDA) are the types of CHDs that are commonly seen in individuals with this condition [13].
(3) **Trisomy 13**: The presence of an extra chromosome 13, called Patau’s syndrome, is associated with congenital heart defects, such as ASDs, VSDs, PDA, and cardiac malpositions such as dextrocardia.
(4) **Turner’s syndrome:** A CHD occurs in 20 to 50% of Turner’s syndrome births. Approximately 10% of the affected cases display a clinically evident heart defect, while the defect in another 10% of cases can be detected by echocardiography. VSDs, coarctation of aorta (CoA), mitral valve prolapse, bicuspid aortic valve, and hypoplastic left heart (HLH) are commonly seen.
(5) **DiGeorge syndrome:** DiGeorge syndrome, one of the most common abnormal chromosome structure syndromes, is characterized by a deletion at 22q11. Cardiac malformations are seen in 80%–100% of individuals with this deletion, including VSDs, arch abnormalities, and tetralogy of Fallot (TOF).
(6) **Williams-Beuren syndrome:** A deletion at 7q11.23, which causes the “Williams-Beuren syndrome”, is also largely associated with congenital malformations such as supravalvar aortic stenosis (SVAS), pulmonary artery sling (PAS), and multiple arterial stenoses.
(7) **Alagille syndrome:** Alagille syndrome, a multisystem disorder involving the heart, liver, face, and skeleton, is characterized by mutations in the JAG1 and NOTCH genes. These genes are critical components of the NOTCH signaling pathway, which regulates various developmental processes. This pathway is involved in the morphogenesis of the heart and the partitioning of the heart by the left-right axis [14]. Ninety percent of individuals with this condition exhibit phenotypes characteristic of peripheral pulmonary hypoplasia, pulmonary stenosis, and TOF [15].
(8) **Char syndrome:** Char syndrome, which is characterized by mutations in the TFAP2B (transcription factor activating enhancer binding protein 2 beta) gene, is usually accompanied by patent ductus arteriosus (PDA).
(9) **Tetrasomy 22q:** This syndrome is called the cat eye syndrome and is characterized by iris colobomata, ear tags, and imperforate anus. Thirty percent of the affected individuals have congenital heart defects, with total anomalous pulmonary venous drainage being the chief problem.
(10) **Chromosome duplication and deletion syndromes:** Macro-deletion syndromes, such as 3q, 4q, 5p, 8p, 9p, 11q, 13q, 18p, and 18q, are commonly associated with congenital heart defects. These defects are also seen in duplication syndromes such as 1p, 2p, 2q, 5p, 8p, 13q, and 16q. Affected children usually have a combination of these deletions and duplications. Examples include the following:
  (a) **Deletion 22q11.2 syndrome**: This syndrome has an incidence of 1 in 4500 live births. It is sporadic in 90% of the cases. The implicated chromosomal region is estimated to encompass close to 30 genes. Three major syndromes are involved with this deletion, namely, DiGeorge syndrome, velo-cardio facial syndrome (VCFS), and conotruncal anomaly face syndrome (CTAFS). TOF, VSDs, and aortic arch anomalies are the common CHDs seen with this condition.
  (b) **1p36 deletion**: Mental retardation, hearing loss, orofacial abnormalities, and microcephaly are several defining traits of this syndrome. Congenital heart defects are seen in 43%–70% of individuals with this syndrome. The most commonly seen defects include PDA, noncompaction, and cardiomyopathy.

**Table 1:** Chromosomal disorders that cause congenital heart diseases.

Disorder	Description	Associated Cardiac Abnormalities
Trisomy 21 (Down’s syndrome)	Extra chromosome 21	AVSD 9
Trisomy 18 (Edward’s syndrome)	Extra chromosome 18	ASD, VSD, PDA
Trisomy 13 (Patau’s syndrome)	Extra chromosome 13	ASD, VSD, PDA
Monosomy X (Turner’s syndrome)	Single X chromosome in females	VSD, CoA, Hypoplastic left heart syndrome (HLHS), Bicuspid Aortic Valve Disease (BAVD)
DiGeorge’s syndrome	Deletion at chromosome 22q11	VSD, TOF, arch abnormalities
William Beuren syndrome	Deletion at chromosome 7q11.23	SVAS, PAS, multiple arterial stenosis
Alagille syndrome	Multiple gene mutations	Peripheral pulmonary hyperplasia, PS, TOF
Char syndrome	Mutations in the TFAP2B gene	PDA
Tetrasomy 22q	Multiple gene mutations	Total anomalous pulmonary venous drainage
Deletion 22q11.2	Chromosome structural anomaly	TOF, VSD, aortic arch anomalies
Deletion 1p36	Chromosome structural anomaly	PDA, cardiomyopathy

### 3.2. Environmental Factors

Non-genetic causes of CHDs include environmental teratogens like polychlorinated biphenyls and pesticides, [16] maternal exposure to alcohol and anti-seizure medications, and infectious agents like rubella [17]. Despite measures being taken to combat the effects of these factors, the array of non-genetic causes has both increased and diversified. Studies have shown that there is a fivefold increase in risk of cardiac malformations in infants born to diabetic mothers. Maternal intake of retinoic acid during the first trimester, poorly controlled diabetes, and untreated phenylketonuria have been shown to be associated with an increased risk of tetralogy of fallot [18,19]. Other emerging risk factors for CHDs include maternal use of anti-retroviral medication and factors such as obesity.

### 3.3. Isolated CHDs

Isolated CHDs are caused by point mutations in genes that are directly or indirectly involved in cardiac morphogenesis. The mechanism by which these genes perturb heart development involves haploinsufficiency or reduction in the dosage of encoded proteins. This can occur by inactivating genes by frameshift or nonsense mutations; altering gene expression by mutations in the non-coding, regulatory regions; or coding for non-functional proteins through missense mutations. These affected genes are involved in one of the following: transcriptional regulation, signal transduction, or encoding cardiac structural proteins.

- **Genes associated with laterality defects:** The heart is the first organ to break the bilateral symmetry of the developing embryo. Crosstalk among signaling pathways like Notch, Nodal, Hedgehog, FGF, and BMP is involved in establishing the left-right symmetry during early embryogenesis. The ZIC3 gene, which encodes the zinc finger transcription factor protein, is one of the upstream players in the Nodal pathway. Mutations in this gene are associated with cardiovascular defects such as transposition of the great arteries (TGA), interrupted aortic arch (IAA), and VSDs along with other developmental abnormalities. CITED2 (Cbp/p300-interacting transactivator, with Glu/Asp-rich carboxy-terminal domain 2) is a transcriptional co-activator that codes for c-AMP-responsive element binding protein. Mutations in this gene are associated with ASD and VSD phenotypes [20]. FOXH1 (forkhead activin signal transducer) is a transcription factor found at the intersection of BMP and Nodal signaling that is involved in establishing left-right symmetry. Absence of an outflow tract and a right ventricle are commonly seen in individuals with mutations in this gene [21]. TOF and TGA are the two most frequently associated CHDs with FOXH1 gene mutations.
- **Genes involved in signaling pathways:** Cardiac development results from spatial and temporal co-ordination of a number of signaling pathways. The NOTCH signaling pathway is involved in left-right axis partitioning and cardiac morphogenesis. JAG1 and its ligand NOTCH1 are implicated in patients with CHD. Mutations in NOTCH1 present as malfunctions of the aortic valve. The CHD phenotypes seen with these mutations include bicuspid aortic valve (BAV), aortic stenosis (AS), CoA, and HLH syndrome.
- **Genes encoding cardiac structural proteins:** These genes constitute the smallest category and least definitive monogenic cause of CHDs. Mutations in the MYH6 gene (myosin heavy chain 6) have been commonly linked to the following CHD phenotypes: ASD, AS, TGA, and patent foramen ovale (PFO). MHY6 gene expression is regulated by transcription factors like GATA4 and TBX5. Mutations in another structural protein, ACTC (alpha cardiac actin), have been seen in cases of ASD.
- **Genes encoding chromatin modifiers:** Modifying chromatin structure is a critical regulatory mechanism for gene expression during heart development. Genes, such as the HHE gene, involved in H2K4 or H3K27 histone modification, are mutated in patients with congenital heart defects.
- **Genes encoding cardiac transcription factors:** Cardiac development is regulated by various transcriptional circuits, and central to these is a set of core transcriptional factors that includes NKX2-5, GATA4, and TBX5. Transcription factors have emerged as prime factors that cause CHDs, with the NKX2-5 gene topping the list.

## 4. Mutations in the NKX2-5 Gene

NKX2-5 belongs to the NK2 family of homeobox genes and is a homolog of the tinman gene found in *Drosophila melanogaster*. It functions as a key regulator in cardiac morphogenesis, regulating the transcription of various genes involved in the process. Mutations in the Tinman in Drosophila embryos results in the loss of heart formation. Studies in murine models and humans have shown consistent expression of the NKX2-5 gene only in the heart, thereby establishing its function in human heart development. While NKX2-5 knockout mice showed early embryonic lethality, mice with loss of one copy of the gene exhibited an array of cardiac abnormalities such as, scattering of AV bundle, reduced HIS and AV node cellular density and abnormal levels of gap junction proteins. More recently, the NKX2-5 gene has been seen to be involved in postnatal cardioprotection. However, its role in enabling myocytes enduring cytotoxic stress needs to be further established.

Mutations in the homeodomain protein NKX2-5 result in a huge number of CHDs, including ASD, VSD, TOF, HLH, CoA, TGA, Double Outlet Right Ventricle (DORV), IAA, and cardiac outflow tract (OFT) defects (Table 2). NKX2-5 mutations mostly result in septal defects and atrio-ventricular conduction defects [22]. While initial studies primarily involved analyzing the exonic regions of the gene, the focus of recent research has shifted to exploring the promoter regions and analyzing intronic variants. Sequencing the NKX2-5 coding region of 26 individuals presenting with an atrioventricular conduction block with or without other CHDs like ASDs and VSDs identified seven novel mutations, including four missense mutations. Three missense mutations (Asn188Lys, Arg189Gly, and Tyr191Cys) were predicted to cause codon changes in the homeodomain, while the fourth (Arg25Cys) was predicted to change a codon in the 5′ coding region. Two other single nucleotide changes (Gln149ter and Tyr259ter) were predicted to introduce a termination site into the homeodomain and the 3′ coding region, respectively. One mutation altered the first nucleotide of the intron (lnt 1DSG + 1T). In addition, an A172G polymorphism was identified.

**Table 2:** List of NKX2-5 mutations reported in previous studies.

Mutation		CHD Phenotype	Reference
Nucleotide Change	Amino Acid Change		
**TN domain**			
A44T	Lys15Ile	Secundum ASD	[23]
A232G	Asn19Ser	VSD	[24]
**Homeodomain**			
T607C	Leu144Pro	ASD, AVSD	[24]
G614T	146Ser	ASD, AVSD	[24]
C554T	Gln149ter	ASD, VSD	[22]
T629C	151Tyr	ASD, VSD, AVSD	[24]
A677G	167Glu	VSD	[24]
C680T	168Arg	VSD	[24]
C618T	Gln170ter	Secundum ASD, AV Block	[20]
A704G	176Lys	ASD, AVSD	[24]
C709T	Thr178Met	VSD	[24]
C642T	Thr178Met	ASD with HLHS	[25]
C533T	Thr178Met	AV block, ASD	[25]
G543A	181Gln	AVSD	[26]
A723G	Lys183Glu	ASD, AVSD	[24]
G554T	Trp185Leu	ASD, VSD	[27]
C735T	Gln187Ter	VSD	[28]
C673A	Asn188Lys	ASD	[22]
C674G	Arg189Gly	ASD	[22]
C568T	Arg190Cys	ASD	[25]
A681G	Tyr191Cys	ASD, VSD	[22]
A751C	Lys192Thr	VSD	[24]
A751G	Lys192Arg	VSD	[24]
A757G	Lys194Arg	VSD	[24]
498-499insC	-	ASD, AV block	[27]
605-606delTG	-	AV block	[27]
**NK2-domain**			
C646T	Arg216Cys	Stenosis	[29]
C656T	Ala219Val	Atresia, TOF	[29,30]
G852A	Asp226Asn	VSD	[28]
**Other regions**			
T196C	Leu7Pro	AVSD	[24]
A239G	21Glu		[24]
G61C	Glu21Gln	TOF, Stenosis	[29,30]
A65C	Gln22Pro	TOF	[30]
A65G	Glu22Arg	ASD	[31]
C249T	Arg25Cys	VSD, TOF	[22,28]
C73T	Arg25Cys	Interrupted aortic arch, TA, TOF, HLHS, stenosis, atresia	[29,30]
T309C	Ser45Pro	VSD	[24]
T327C	Phe51Leu	VSD	[24]
C188T	Ala63Val	l-TGA	[30]
T382C	Leu69Pro	VSD	[24]
C406T	Pro77Leu	VSD	[24]
T516A	Cys114Ser	ASD, AVSD	[24]
T516C	Cys114Arg	ASD, VSD, AVSD	[24]
A529G	Lys118Arg	ASD, VSD	[24]
G355T	Ala119Ser	HLHS	[26]
C356A	Ala119Glu	AVSD	[26]
A547G	Lys124Arg	VSD	[24]
A553T	Glu126Val	ASD, VSD, AVSD	[24]
C380A	Ala127Glu	Secundum ASD	[30]
C560T	128Asp	ASD, AVSD	[24]
C573T	Pro133Ser	VSD	[24]
G579A	Ala135Thr	ASD, AVSD	[24]
C701T	Gln198ter	Secundum ASD, AV Block	[20]
T779C	201Thr	VSD	[24]
T790A	Val205Glu	VSD	[24]
InsTCCCT701	D235AFSter	Secundum ASD; First-degree AV block	[30]
C902G	242Gly	VSD	[24]
T918C	Tyr248His	VSD	[24]
C886A	Tyr259ter	ASD, VSD	[22]
T995C	273Ala	ASD, VSD, AVSD	[24]
C823A	Pro275Thr	CoA	[24]
T1011C	Ser279Pro	VSD	[24]
C1012T	Ser279Phe	VSD	[24]
C1018T	Ala281Val	ASD, VSD, AVSD	[24]
C1033T	Ala286Val	ASD, VSD, AVSD	[24]
C1034T	286Ala	ASD, AVSD	[24]
delAAC871	del291Asn	DORV	[30]
A1056C	Asn294His	ASD	[24]
A1072G	Asp299Gly	ASD, VSD, AVSD	[24]
T1079C	301Asn	VSD	[24]
A1089G	Ser305Gly	VSD	[24]
A1118G	314Gly	ASD, VSD, AVSD	[24]
G1134A	Gly320Ser	ASD, VSD, AVSD	[24]
G1141A	Arg322Gln	VSD	[24]
A1142G	322Arg	ASD, VSD, AVSD	[25]
G967A	Ala323Thr	TOF	[30]
C113T	-	VSD	[25]
C141T	-	VSD	[25]
G1156A	-	ASD, VSD, AVSD	[25]
215-221-delAGCTGGG	-	ASD, VSD, Heterotaxia, MV Abnormality	[32]
T1149C	STOP-Gln	ASD, VSD, AVSD	[29]
Int1DSG+1T	-	AV block	[22]

Functional analysis of three NKX2.5 gene mutations, namely Gln170ter, Thr178Met, and Gln198ter, showed that Gln170ter introduced a stop codon resulting in premature termination of homeodomain translation, Thr178Met caused a non-synonymous substitution in the homeodomain region, and Gln198ter introduced a stop codon immediately after the homeodomain [33]. Examining the transcriptional activity of the ANP promoter (atrial natriuretic peptide), which is activated in synergy with NKX2.5 and GATA-4, and DNA binding with GATA-4 revealed that ANP promoter activity was enhanced by Gln198ter but was inhibited by Gln170ter and Thr178Met. Thus, despite conferring the same cardiac phenotype, transactivation ability and DNA binding ability were different among the three mutants.

In addition to the existing conundrum, some studies identified mutations that are not completely penetrant, unlike the previously reported homeodomain mutations [29]. Subsequent studies analyzed non-homeodomain regions of the NKX2-5 gene in an attempt to discover other significant variations that may contribute to the development of cardiac anomalies. A 2003 study [30] analyzed the genomic DNA of 608 individuals with different CHDs using direct sequencing or conformation-sensitive gel electrophoresis. Twelve distinct mutations were identified in 18 of the 608 individuals evaluated. Moreover, two of the 18 individuals were known to have at least one relative with a CHD. None of these mutations observed were located in the homeodomain. Eleven of the mutations were missense nucleotide substitutions or in-frame deletions, while one was a five-nucleotide insertion predicted to cause premature termination of translation. These findings highlight the role of the non-homeodomain regions of the NKX2-5 gene in congenital heart disease.

In addition to single mutations, specific haplotypes comprising multiple mutations have also been identified in affected individuals [28]. In addition, non-synonymous mutations have been seen in other transcription factor genes, such as GATA4, TBX5, and HAND1. Surprisingly, these mutations were found only in the cardiac tissue sections of patients and not in their peripheral blood samples. This suggests that NKX2-5 gene mutations may be mosaic in nature and may not be detected in a simple peripheral blood analysis. This mosaicism also implicates somatic mutations as novel factors contributing to congenital heart disease.

The NKX2-5 gene is also commonly implicated in familial CHDs. A familial screen of five female members across three generations who were affected by AV conduction block associated with various CHD types revealed a novel heterozygous mutation (c.325G > T). This mutation was found to co-segregate with the disease condition and was predicted to introduce a stop codon at amino acid position 109, resulting in a truncated protein with no functional significance [34]. The subsequent loss of NKX2-5 function and haploinsufficiency paves the way for the development of CHDs. Mutations in the NKX2-5 gene are frequently seen in congenital ASDs [35], with mutations co-segregating with ASDs in the families that have complete penetrance.

**Figure 2:**
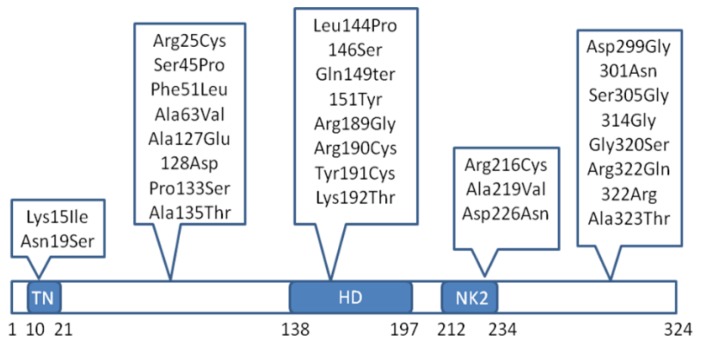
Spectrum of some significant NKX2-5 gene mutations implicated in congenital heart defects. TN—tin-man domain/transcriptional repression domain, HD—homeodomain.

More recent studies have shifted their focus to the regulatory regions of the NKX2-5 gene [36]. Functional analysis of these non-coding variants revealed enhanced transcriptional activity of the NKX2-5 gene promoter, thereby altering the NKX2-5 expression levels and hence the gene regulatory network governing cardiac morphogenesis. There is also wide speculation regarding the enhanced gene regulatory effects of NKX2-5 mutations. It has been proposed that these mutants can go off-track and bind to a number of non-specific genes, thereby allowing co-factors to induce a stronger effect than usual [37]. This phenomenon explains the variation seen in the severity of the disease in affected individuals. To date, approximately 50 different mutations have been identified in the NKX2-5 gene, including missense mutations, synonymous mutations, and nonsense mutations. Figure 2 depicts the location of some of these mutations.

Very rarely, mutations in other transcription factors such as GATA4 have been seen to occur in the absence of a mutated NKX-5 gene. The GATA4 protein regulates genes involved in myocardial differentiation and function. Haploinsufficiency of GATA4 is seen in patients with interstitial deletions in 8p23.1; hence, a correct GATA4 dosage is needed for normal cardiogenesis. CHD phenotypes associated with GATA4 mutations include ASDs, PS, VSDs, TOF, AVSD, and Partial anomalous pulmonary venous return (PAPVR).

## 5. Significance of Genetic Testing and Counseling

The worldwide incidence of congenital heart defects is reported to be 1–17.5 per 1000 live births. Late detection of disease preventing prompt medical intervention is the main reason for such a high incidence. Renowned bodies such as the American Heart Association and the Canadian Cardiovascular society recommend genetic counseling and testing for all CHD patients with one or more genetic risk factors, with or without the presence of syndromic characteristics [38,39]. The research performed to establish the etiology of congenital heart defects implicates aberrations in genes, with the NKX2-5 gene being the prime suspect. Mutations in the NKX2-5 gene are primarily responsible for the development of ASDs, tetralogy of fallot, and ventricular septal defects.

Screening for NKX2-5 gene mutations can provide early identification of a variety of congenital heart defects, including those mentioned above. As NKX2-5 mutations exhibit common inheritance patterns, screening could also identify family members who may also be at risk.

While ASDs in children are easily manageable, they only manifest in adults after causing irreversible damage to the heart and lungs [32]. Approximately 20% of ASD individuals are likely to develop an atrio-ventricular block later in life and may require additional surgical intervention [25]. Severe AV cases require frequent lifelong monitoring post-treatment. Early identification may thus facilitate optimal treatment planning.

## 6. Conclusions

Cardiogenesis is a complicated process, comprising of numerous intricate pathways, where NKX2-5 plays a pivotal role. Aberrations in the NKX2-5 transcription factor have been reported in various types of congenital heart defects and are spread across various domains on the gene. Functional studies have shed light on the mechanism of action of these mutations but more analysis will be needed to understand the entire picture of cardiac malformation. While the most commonly accepted hypothesis remains reduced levels of all molecules involved in heart development, other models such as the two-hit hypothesis in inherited cases are gaining importance. Robust genomic analysis will be the key to unlocking the complexity of congenital heart diseases.

## 7. Future Perspectives

The genetics of CHDs is complex, and we understand only approximately a third of the underlying molecular machinery. Currently, importance is being given to determining the aberrant expression regulation of cardiac progenitors, and research into the role mi-RNA in cardiogenesis is at the forefront of these efforts. In addition, with the successful development of techniques such as genome-wide association studies and high-throughput sequencing, it is possible to determine the various genetic variations responsible for a cardiac phenotype in an individual.

In cases of CHDs, genetic analysis and counseling should be mandatory to provide technical and psychological support to the affected individuals.
